# Effects of oral administration of 5 immunosuppressive agents on activated T‐cell cytokine expression in healthy dogs

**DOI:** 10.1111/jvim.15729

**Published:** 2020-02-13

**Authors:** Todd M. Archer, Charlee Mulligan, Lakshmi Narayanan, Caitlin Riggs, Claire Fellman, John M. Thomason, Robert W. Wills, Dawn M. Boothe, Crisanta Cruz‐Espindola, Roy Harmon, Andrew J. Mackin

**Affiliations:** ^1^ Department of Clinical Sciences College of Veterinary Medicine, Mississippi State University Starkville Mississippi; ^2^ Department of Population and Preventative Medicine College of Veterinary Medicine, Mississippi State University Starkville Mississippi; ^3^ Department of Anatomy, Physiology and Pharmacology College of Veterinary Medicine, Auburn University Auburn Alabama; ^4^ Department of Clinical Sciences Tufts University North MA

**Keywords:** azathioprine, cyclosporine, interferon gamma, interleukin 2, leflunomide, mycophenolate mofetil, prednisone [Correction added on March 16, 2020, after first online publication: See Appendix S1 for correction details.]

## Abstract

**Background:**

Dogs are often adminstered >1 immunosuppressive medication when treating immune‐mediated diseases, and determining whether these different medications affect IL‐2 expression would be useful when performing pharmacodynamic monitoring during cyclosporine therapy.

**Hypothesis/Objectives:**

To determine the effects of 5 medications (prednisone, cyclosporine, azathioprine, mycophenolate mofetil, and leflunomide) on activated T‐cell expression of the cytokines IL‐2 and interferon‐gamma (IFN‐γ).

**Animals:**

Eight healthy dogs.

**Methods:**

Randomized, cross‐over study comparing values before and after treatment, and comparing values after treatment among drugs. Dogs were administered each drug at standard oral doses for 1 week, with a washout of at least 21 days. Activated T‐cell expression of IL‐2 and IFN‐γ mRNA was measured by quantitative reverse transcription polymerase chain reaction. Blood drug concentrations were measured for cyclosporine, mycophenolate, and leflunomide metabolites.

**Results:**

Least squares means (with 95% confidence interval) before treatment for IL‐2 (2.91 [2.32‐3.50] ΔCt) and IFN‐γ (2.33 [1.66‐3.00 ΔCt]) values were significantly lower (both *P* < .001) than values after treatment (10.75 [10.16‐11.34] and 10.79 [10.11‐11.46] ΔCt, respectively) with cyclosporine. Similarly, least squares means before treatment for IL‐2 (1.55 [1.07‐2.02] ΔCt) and IFN‐γ (2.62 [2.32‐2.92] ΔCt) values were significantly lower (both *P* < .001) than values after treatment (3.55 [3.06‐4.00] and 5.22 [4.92‐5.52] ΔCt, respectively) with prednisone. Comparing delta cycle threshold values after treatment among drugs, cyclosporine was significantly different than prednisone (IL‐2 and IFN‐γ both *P* < .001), with cyclosporine more suppressive than prednisone.

**Conclusions and Clinical Importance:**

Prednisone and cyclosporine both affected expression of IL‐2 and IFN‐γ, suggesting that both have the ability to influence results when utilizing pharmacodynamic monitoring of cyclosporine treatment.

**Abbreviations**
ΔCt: delta cycle threshold
HPLC: high‐performance liquid chromatography
IFN‐γ: interferon‐gamma
IL‐2: interleukin‐2
LOQ: limit of quantification
RT‐qPCR: quantitative reverse transcription polymerase chain reaction
UV: ultraviolet

## 1 INTRODUCTION

Immune‐mediated disorders of dogs are commonly treated with 1 or more immunosuppressive agents, often a glucocorticoid in addition to a second immunosuppressive agent such as cyclosporine, azathioprine, mycophenolate mofetil, or leflunomide.^1^ Glucocorticoids such as prednisone are a mainstay in the treatment of immune‐mediated disorders, with multiple mechanisms of action involving both the humoral and cell‐mediated arms of immunity.^1^ Cyclosporine has a specific mechanism of action, which is inhibition of the enzyme calcineurin in lymphocytes, leading to impaired lymphocyte function by suppression of nuclear factor of activated T‐cell‐regulated cytokines such as interleukin‐2 (IL‐2) and interferon‐gamma (IFN‐γ).^1^ Mycophenolate mofetil and azathioprine are both purine synthesis inhibitors, whereas leflunomide is a pyrimidine synthesis inhibitor, and the resultant impairment of nucleotide synthesis suppresses multiple DNA‐ and RNA‐dependent functions of B‐ and T‐cells.^1^ Cyclosporine's effects on the T‐cell cytokines IL‐2 and IFN‐γ in dogs are well established,^2^, ^3^, ^4^ but the effects of standard doses of other oral immunosuppressive medications have not been thoroughly investigated.

In dogs receiving immunosuppressive treatment, therapeutic drug monitoring through the measurement of blood concentrations, the use of pharmacodynamic assays, or both can be used to ensure appropriate drug doses, particularly when there is individual dog‐to‐dog variability in drug metabolism and a narrow therapeutic window between ineffective and toxic doses. Currently, there are no commercially available assays for measuring blood concentrations of prednisone, mycophenolate mofetil, or azathioprine in dogs, although concentrations of mycophenolic acid (the active metabolite of mycophenolate mofetil) have been measured in an experimental setting. In North America, there are currently commercially available assays for measuring cyclosporine and teriflunomide (the active metabolite of leflunomide) blood concentrations. There is also a commercially available pharmacodynamic assay to evaluate the immunosuppressive effects of cyclosporine in dog via measurement of activated T‐cell production of IL‐2 and IFN‐γ. This assay was developed and validated specifically for cyclosporine^5^ and has not been evaluated with other immunosuppressive drugs. Many dogs receive >1 immunosuppressive medication when treating immune‐mediated diseases, and it is unknown if any of the other common immunosuppressive medications might also affect T‐cell production of IL‐2 and IFN‐γ. Identifying the effects of the most common immunosuppressive medications on T‐cell cytokine expression would be helpful in better understanding their effects on the immune system, as well as determining the potential influence of the concurrent administration of other immunosuppressive drugs on pharmacodynamic assay results in dogs receiving cyclosporine.

The goal of this study was to identify the effects of the oral administration, at immunosuppressive doses, of 5 common immunosuppressive medications (prednisone, cyclosporine, azathioprine, mycophenolate mofetil, and leflunomide) on the expression of the T‐cell cytokines IL‐2 and IFN‐γ in dogs. Our hypotheses were that cyclosporine and prednisone would suppress cytokine gene expression because they are known to affect transcription factors, with cyclosporine having the greatest effect, and that the proliferation inhibitors azathioprine, mycophenolate mofetil, and leflunomide would not have a significant effect.

## 2 MATERIAL AND METHODS

### 2.1 Animals

Eight healthy Walker hound dogs were used in this study. Four dogs were intact females and 4 were neutered males, with a median age of 2 years (range, 1.3‐7.3 years). Typically, 6‐8 dogs are used for standard pharmacokinetic and pharmacodynamic studies, as described by Riviere,^6^ and published by others.^2^, ^3^, ^7^, ^8^, ^9^ Dogs were deemed healthy by detection of no abnormalities on physical examination and a minimum data base (including a complete blood count, serum biochemistry, urinalysis) as well as heartworm testing. The dogs were not exposed to any medications or vaccines for at least 1 month before the initiation of the study. Animal use was approved by the Mississippi State University College of Veterinary Medicine Institutional Animal Care and Use Committee and followed the requirements of a facility accredited by the American Association for Accreditation of Laboratory Animal Care.

### 2.2 Drug administration and blood collection

In a 5‐way, randomized, cross‐over study, the dogs were administered either prednisone (West‐Ward Pharmaceutical Corp., Eatontown, New Jersey) (2.0 ± 0.1 mg/kg, mean ± SD, PO, q24h), azathioprine (Mylan Pharmaceuticals Inc, Morgantown, West Virginia) (1.8 ± 0.1 mg/kg, PO, q24h), cyclosporine (Atopica; Novartis Animal Health, Greensboro, North Carolina) (10.3 ± 0.2 mg/kg, PO, q12h), mycophenolate mofetil (West‐Ward Pharmaceutical Corp.) (9.9 ± 0.4 mg/kg, PO, q12h), or leflunomide (Trigen Laboratories, LLC, Sayreville, New Jersey) (4.0 ± 0.2 mg/kg, PO, q24h). With each medication, drugs were administered for 7 days, followed by a minimum recovery period of 21 days between dosing with another medication. In previous studies of cyclosporine, maximal changes in cytokine expression were seen within 1 week of commencing the drug, and cytokine expression had returned to values before treatment within 2 weeks of discontinuing the medications.^2^, ^4^, ^9^ After each recovery period, the dogs switched groups, and the study was continued until all dogs had received all medications.

Before drug administration, 3 mL of blood was collected from each dog in a heparinized tube for baseline quantitative reverse transcription polymerase chain reaction (RT‐qPCR) analysis for the assessment of IL‐2 and INF‐γ. At the end of each treatment period, blood was again collected in a heparinized tube from each dog, 2 hours after the morning drug dose was administered, for repeat RT‐qPCR analysis. At the same time point (2 hours after the final morning pill), blood was collected to measure peak blood cyclosporine concentrations. Additionally, within minutes before administration of the final morning drug dose, blood was collected to measure trough blood concentrations of the active metabolites of mycophenolate mofetil and leflunomide (mycophenolic acid and teriflunomide, respectively).

### 2.3 Blood incubation, T‐cell activation, and RNA extraction

All whole blood samples were activated before RNA extraction with 12.5 ng/mL of phorbol myristate acetate (Sigma, St. Louis, Missouri) and 0.8 μM of ionomycin (Sigma), as previously described.^5^ All samples were then incubated for 5 hours at 37°C and 5% CO_2_.

RNA was extracted by using a previously published protocol.^5^ Total RNA was isolated from 1 mL of heparinized whole blood by using a QIAamp Whole Blood RNA Mini Kit (Qiagen, Valencia, California, Cat. No. 52304). Genomic DNA was removed from the samples according to the manufacturer's instructions of an on‐column DNase (27.27 Kunitz units) treatment (Qiagen, Valencia, California, Cat. No. 79254). Samples were then stored at −80°C until further analysis.

### 2.4 Cytokine gene expression quantification

The concentration and purity of the RNA were estimated by a Nanodrop ND‐1000 spectrophotometer with ND‐1000 V3.3.0 software (NanoDrop Technologies, Wilmington Delaware). A SuperScript III Platinum SYBR Green One‐Step kit with Rox as a reference dye (Invitrogen, Grand Island, New York, Cat no. 11736‐059) was used to quantify expression of the genes of interest (IL‐2 and IFN‐γ) and the expression of the reference gene GAPDH. Primers were based on previously published GenBank sequences published by Kobayashi et al.^10^ All reactions were performed in a 96‐well format on a Stratagene Mx3005P Multiplex Quantitative PCR system (Agilent Technologies, Santa Clara, CA) and analyzed with MxPro software. The RT‐qPCR reaction was performed with a final volume of 20 μL containing a total of 30 ng of template RNA and 200 nM of each primer. The following thermal cycling parameters were used: 50°C for 3 minutes, 95°C for 5 minutes, then 40 cycles of 95°C for 15 seconds and 60°C for 30 seconds. Melting curve analysis performed. All samples were run in triplicate, whereas nontemplate controls were run in duplicate. Delta cycle threshold (ΔCt) values were compared against each other for pretreatment activated baseline samples as well as samples on Day 7 of drug treatment, where ΔCt = Ct_GOI_ ‐ Ct_norm_, in which GOI is the gene of interest and norm is the reference gene. The 2^–ΔΔCt^ method was used to determine the relative change in expression by using GAPDH as a reference gene where ΔΔCt = (Ct_GOI_ – Ct_norm_)_treated_ – (Ct_GOI_ – Ct_norm_)_pretreatment_.^11^ Cytokine gene expression was presented as a percentage compared to pretreatment‐activated baseline samples for samples collected on Day 7 of drug treatment, where pretreatment‐activated baseline samples represented 100% gene expression for IL‐2 and IFN‐γ. Relative expression was calculated by using the formula: (2^–ΔΔCt^) * 100%. [Correction added on March 16, 2020, after first online publication: See Appendix S1 for correction details.]

### 2.5 Blood drug concentrations

Whole blood samples (Ethylenediaminetetraacetic acid [EDTA] anticoagulant) collected 2 hours after the final morning drug dose were submitted to the Auburn University Clinical Pharmacology Laboratory for measurement of peak blood cyclosporine concentrations. Plasma derived from whole blood (EDTA anticoagulant) collected within minutes before the final morning dose was also submitted to the same laboratory for measurement of trough mycophenolic acid and teriflunomide concentrations.

Canine plasma was analyzed for the active leflunomide metabolite, teriflunomide (A77‐1726), by high‐performance liquid chromatography (HPLC) with ultraviolet (UV) detection. The HPLC system consisted of a Waters 717 plus autosampler, Waters 1525 Binary pump and a 2487 UV‐visible detector (Waters Corporation, Milford, MA).^12^, ^13^ Separation was achieved with a Luna C18, 5 μm, 150 × 4.6 mm column (Phenomenex Inc, Torrance, CA).^12^, ^13^, ^14^ The mobile phase consisted of 0.05 M potassium phosphate monobasic, pH 3.0,^12^, ^13^ and acetonitrile (VWR, Radnor, PA),^3^ with the flow rate set to 1 mL/min.^12^ The standard curve ranged from 2.5 to 200 μg/mL, and was generated by spiking canine plasma with known amounts of teriflunomide (VWR, Radnor, PA) reference standard and accepted if the coefficient of determination (*r* ^2^) was at least .999 and the predicted concentrations were within 10% of the actual concentrations. Briefly, 500 μL of acetonitrile were added to tubes containing 250 μL of plasma sample.^14^ The contents of each tube were mixed vigorously through vortexing, then subjected to centrifugation for 10 minutes at 3000 rpm (1900*g*).^12^, ^14^ The volume of clear supernatant injected into the column was 20 μL.^14^ The retention time for teriflunomide was 4.5 minutes and UV absorbance was monitored at 302 nm^2^. The linear correlation coefficient for teriflunomide was .9995. The limit of detection and the limit of quantification (LOQ) for teriflunomide were 1.0 and 2.5 μg/mL, respectively. The accuracy (% recovery) for teriflunomide plasma controls samples with concentrations of 2.5, 5, 10, 50, 100, and 200 μg/mL was 92.8, 102.2, 106.6, 103.3, 94.3, and 98.8%, respectively. The precision (CV%) for teriflunomide plasma control samples with concentrations of 2.5, 5, 10, 50, 100, and 200 μg/mL was 3.3, 1.5, 1.9, 3.4, 2.5, and 4.5% respectively.

Whole blood concentrations of cyclosporine A were measured by using an automated immunoassay on a Siemens Dimension Xpand Plus integrated chemistry system (Siemens Healthcare Diagnostics Inc, Tarrytown, NY). The operator loaded samples and reagents onto the analyzer and assigned tests for each sample; all subsequent actions were automatically performed by the Dimension system. The assay lysed a sample of blood and the cyclosporine molecules contained in that sample bind to an antibody‐enzyme (β‐galactosidase) conjugate. Chromium dioxide particles coated with cyclosporine were added to bind the excess antibody‐enzyme conjugate, and the mixture was separated magnetically. The fraction of antibody‐enzyme conjugate bound to intrinsic cyclosporine was mixed with chlorophenol red‐β‐d‐galactopyranoside to yield chlorophenol red. Because the product of this hydrolysis reaction has a photometric absorbance peak at 577 nm, the cyclosporine concentration of the sample was quantified by measuring the bichromatic (577 and 700 nm) change in absorbance. The LOQ for cyclosporine was 25 ng/mL. The accuracy (% recovery) for cyclosporine plasma control samples with concentrations of 80, 350, 600, and 1800 ng/mL was 99.6, 96.4, 101.5, and 100.6%, respectively. The precision (CV%) for cyclosporine plasma controls samples with concentrations of 80, 350, 600, and 1800 ng/mL was 10.9, 4.5, 5.4, and 5.1%, respectively.

Plasma mycophenolic acid levels were measured by using a homogeneous particle‐enhanced turbidimetric inhibition immunoassay, automated assay, on the Siemens Dimension Xpand Plus system (Siemens Healthcare Diagnostics Inc). The assay used a synthetic particle‐mycophenolic acid conjugate and monoclonal antibody specific to mycophenolic acid. Competition for antibody between the particle‐conjugated mycophenolic acid and the mycophenolic acid in the plasma sample determined the rate of aggregation; the rate of aggregation was measured with bichromatic (340 and 700 nm) turbidimetric readings and was inversely proportional to the plasma mycophenolic acid concentration. The LOQ was 0.2 μg/mL. The accuracy (% recovery) for mycophenolic acid plasma control samples with concentrations of 2.5, 7.5, and 25 μg/mL was 109.3, 108.4, and 102.7%, respectively. The precision (CV %) for mycophenolic acid plasma controls samples of 2.5, 7.5, and 25 μg/mL was 1.7, 0.6, and 5.3%, respectively. Manufacturer documentation indicates 61% cross‐reactivity with the parent drug (mycophenolate mofetil) and 49% cross‐reactivity with the primary metabolite (7‐*O*‐mycophenolic acid [MPA]‐β‐glucuronide) in spiked samples containing mycophenolic acid concentrations of 0‐5 μg/mL.

### 2.6 Statistical analysis

In separate models for IL‐2 ΔCt and IFN‐γ ΔCt, pretreatment baseline cytokine levels were compared among cycles by using general linear models with PROC MIXED. The effect of sample time and cycle on the cytokine levels was assessed by using mixed model linear regression with PROC MIXED in SAS for Windows 9.4 (SAS Institute, Inc, Cary, NC). Separate models were fit for each drug with IL‐2 ΔCt or IFN‐γ ΔCt as the response variable. In each model, sample time, cycle, and their interaction were included as fixed effects. Dog identity was included as a random effect. If the sample time‐cycle interaction term was not significant, it was removed and the model refits. The effect of the different drugs on the Day 7 cytokine levels was also assessed by using mixed model linear regression. Separate models were fit for IL‐2 ΔCt and IFN‐γ ΔCt. In each model, drug, cycle, and their interaction were included as fixed effects with the cytokine level in pretreatment samples included as a covariate. Dog identity was included as a random effect. If the drug‐cycle interaction was not significant, it was removed and the model refits. Differences in least squares means of cytokine levels were determined for significant fixed effects. The simulation option was used for adjustment of *P* values for multiple comparisons. The distribution of the conditional residuals was evaluated for each outcome to ensure the assumptions of normality and homoscedasticity for the statistical method had been met. An alpha level of .05 was used to determine statistical significance for all methods.

## 3 RESULTS

### 3.1 IL‐2 and IFN‐γ analyses

**Figure 1:**
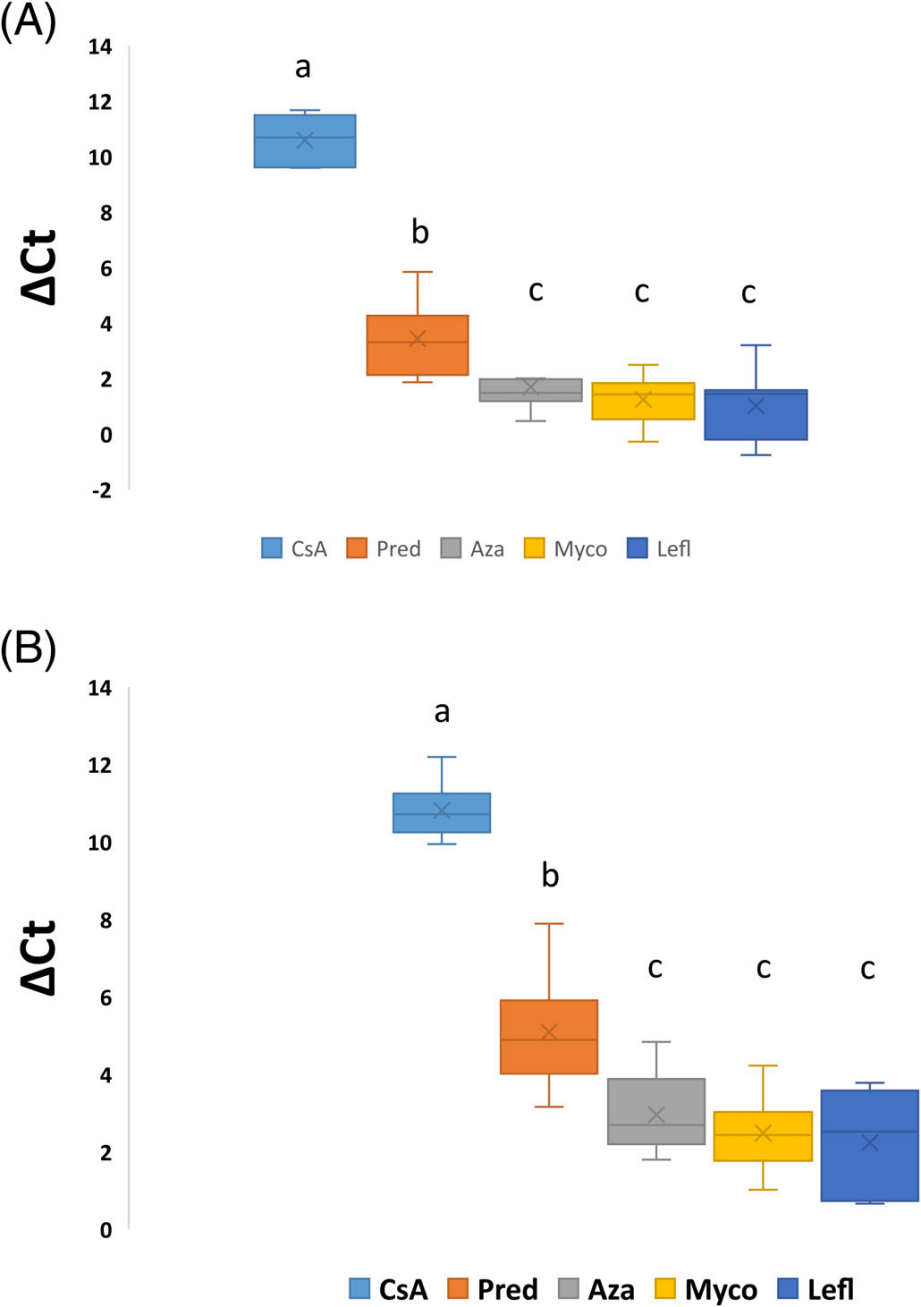
Box plots of activated whole blood IL‐2 (A) and IFN‐γ (B) mRNA expression for 8 healthy Walker hounds after treatment. The effects of administration of 5 different immunosuppressive drugs on activated samples is demonstrated, with expression presented as ΔCt values where ΔCt = Ct_GOI_ – Ct_norm_, in which GOI is the gene of interest and norm is the reference gene. The line within each box denotes the median, the *x* denotes the mean, the box edges represent the first and third quartiles, and the whiskers extend to maximum and minimum values. Samples that share the same letter are not significantly different. Aza, azathioprine; CsA, cyclosporine; IFN‐γ, interferon‐gamma; IL‐2, interleukin‐2; Myco, mycophenolate; Pred, prednisone; Lefl, leflunomide

**Figure 2:**
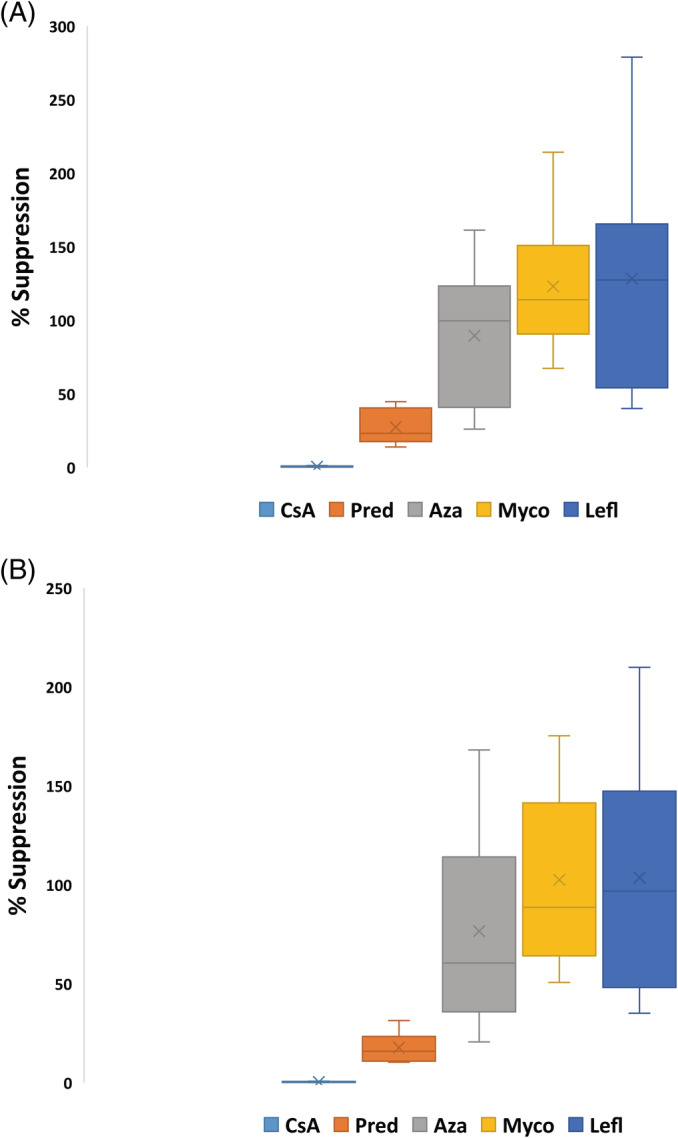
Box plots of activated whole blood IL‐2 (A) and IFN‐γ (B) RNA expression for 8 healthy Walker hounds presented as a percentage of untreated activated baseline samples, in which the untreated activated baseline samples represent 100% cytokine production. The effects of prednisone, cyclosporine, azathioprine, mycophenolate mofetil, and leflunomide on T‐cell cytokine mRNA expression over 7 days of treatment are presented. The line within each box denotes the median, the x denotes the mean, the box edges represent the first and third quartiles, and the whiskers extend to maximum and minimum values. Less than 5% of pretreatment values is clinically categorized by the reference laboratory as marked suppression. Aza, azathioprine; CsA, cyclosporine; IFN‐γ, interferon‐gamma; IL‐2, interleukin‐2; Lefl, leflunomide; Myco, mycophenolate; Pred, prednisone [Correction added on March 16, 2020, after first online publication: See Appendix S1 for correction details.]

Quantitative reverse transcription polymerase chain reaction results are presented in Table 1 and Figure 1 for least squares mean IL‐2 and IFN‐γ ΔCt values and Figure 2 for IL‐2 and IFN‐γ median percent suppression for each drug. Increased ΔCt corresponds to decreased cytokine RNA expression. When comparing the ΔCt values of the pretreatment baseline activated samples to the samples collected on Day 7, there was significant cytokine suppression during cyclosporine (IL‐2 [*P* < .001], IFN‐γ [*P* < .001]) and prednisone (IL‐2 [*P* < .001], IFN‐γ [*P* = .001]) administration. When comparing pretreatment baseline‐activated samples to the samples collected on Day 7 of azathioprine, mycophenolate mofetil, and leflunomide administration, there was no significant differences in cytokine expression (IL‐2: *P* > .2772; IFN‐γ: *P* > .1007). When comparing the ΔCt values on Day 7, there were significant differences between cyclosporine and prednisone (IL‐2 [*P* < .001], IFN‐γ [*P* < .001]), azathioprine (IL‐2 [*P* < .001], IFN‐γ [*P* < .001]), mycophenolate mofetil (IL‐2 [*P* < .001], IFN‐γ [*P* < .001]), and leflunomide (IL‐2 [*P* < .001], IFN‐γ [*P* < .001]). There were significant differences between prednisone and azathioprine (IL‐2 [*P* = .0071], IFN‐γ [*P* = .0006]), mycophenolate mofetil (IL‐2 [*P* = .0002], IFN‐γ [*P* < .001]), and leflunomide (IL‐2 [*P* = .0002], IFN‐γ [*P* < .001]). There were no significant differences among azathioprine, mycophenolate mofetil, and leflunomide groups for IL‐2 (*P* > .5004) and IFN‐γ (*P* > .4055). Significant differences in baseline levels before treatment were not detected among cycles for either IL‐2 ΔCt (*P* = .3334) or IFN‐γ ΔCt (*P* = .1806).

**Table 1:** Least squared mean ΔCt values (for 8 healthy Walker hounds) with 95% confidence interval of baseline‐activated samples before treatment and activated samples after treatment (7th day of drug administration) for prednisone, cyclosporine, azathioprine, mycophenolate mofetil, and leflunomide on T‐cell cytokine mRNA expression, with expression presented as ΔCt values where ΔCt = Ct_GOI_ – Ct_norm_, in which GOI is the gene of interest and norm is the reference gene

	Pred	CsA	Aza	Myco	Lefl
IL‐2					
Before treatment	1.55 (1.074‐2.020)	2.91 (2.319‐3.504)	1.33 (0.315‐2.349)	1.46 (0.800‐2.112)	1.14 (0.437‐1.840)
Treatment	3.55* (3.056‐4.002)	10.75* (10.157‐11.342)	1.72 (0.708‐2.742)	1.24 (0.584‐1.895)	1.04 (0.337‐1.740)
IFN‐γ					
Before treatment	2.62 (2.319‐2.924)	2.33 (1.658‐3.009)	2.34 (1.586‐3.087)	2.46 (2.072‐2.840)	2.15 (1.542‐2.765)
Treatment	5.22* (4.916‐5.521)	10.79* (10.114‐11.465)	3.01 (2.264‐3.766)	2.54 (2.158‐2.926)	2.34 (1.732‐2.955)

Abbreviations: Aza, azathioprine; CsA, cyclosporine; IFN‐γ, interferon‐gamma; IL‐2, interleukin‐2; Lefl, leflunomide; Myco, mycophenolate; Pred, prednisone.

* Significant difference when comparing values before treatment to values after treatment.

Cytokine expression, presented as a percentage of baseline‐activated samples before treatment, is represented in Figure 2. Baseline‐activated samples before treatment represents 100% cytokine production. Samples exposed to cyclosporine showed a marked suppression of cytokine expression after 7 days of drug administration.

### 3.2 Blood drug concentrations

The median peak cyclosporine whole blood concentration was 2364 ng/mL (range, 1671‐3052 ng/mL). For mycophenolic acid trough plasma concentrations, the median value was 2.2 μg/mL (range, 0.3‐5.8 μg/mL). For teriflunomide trough plasma concentrations, the median value was 99.9 μg/mL (range, 77.3‐123.2 μg/mL).

## 4 DISCUSSION

In this study, we utilized a RT‐qPCR assay, validated in dogs, to explore the effects of the administration of 5 common immunosuppressive medications on T‐cell cytokine production. This study demonstrated that the oral administration of cyclosporine and prednisone significantly decreased activated T‐cell expression of IL‐2 and IFN‐γ in healthy dogs, when assessing ΔCt values, but mycophenolate mofetil, azathioprine, and leflunomide did not significantly affect cytokine gene expression. Additionally, there was a greater suppression of IL‐2 and IFN‐γ during cyclosporine administration when compared to prednisone administration.

Immune‐mediated and inflammatory disorders of dogs are commonly treated with ≥1 immunosuppressive agents. In dogs, this often includes a glucocorticoid in addition to a second immunosuppressive agent, not only to target the immune system with 2 medications with differing mechanisms of action, but also to enable earlier tapering of the glucocorticoid to minimize steroid‐related adverse effects.^1^ Prednisone, cyclosporine, mycophenolate mofetil, azathioprine, and leflunomide can all be used in dogs to induce immune suppression.^1^, ^15^ For all these medications, there is a range of recommended dosages for use in dogs, and this study used common starting dosages that are believed to be immunosuppressive in dogs.^1^, ^15^ The cyclosporine dosage chosen for this study was 10 mg/kg PO twice daily, and although this is within the accepted dosage range for initial treatment, it is at the high end of the dosage range. This dose was chosen to reliably induce immune suppression in all dogs. This study revealed that dogs administered cyclosporine at this high dose exhibited marked suppression of cytokine expression after 7 days of drug administration, demonstrating that this assay worked in a manner consistent with previous studies, and confirming that the drug dose used was reliably immunosuppressive.^2^, ^5^ The more common cyclosporine dosage of 5 mg/kg PO twice daily does not reliably induce suppression of IL‐2 and IFN‐γ in all dogs, based on experience from the Clinical Pharmacodynamic Laboratory at Mississippi State University. All other dosages used followed recommended guidelines from the ACVIM consensus statement on the treatment of immune‐mediated hemolytic anemia in dogs.^15^

In dogs with immune‐mediated disorders, there is limited evidence that prednisone provides superior immunosuppression when compared to other immunosuppressive drugs, as randomized prospective clinical trials are lacking. Other immunosuppressive medications are often used in combination with prednisone to suppress the immune system via >1 mechanism of action, as well as to allow for tapering of the dose of prednisone whereas ≥1 other immunosuppressive drugs continue to inhibit the immune system and promote ongoing disease remission.^1^ The effects of most of the immunosuppressive drugs commonly used in dogs on T‐cell IL‐2 and IFN‐γ expression are mostly unknown, except forcyclosporine, which has been documented to markedly suppress these cytokines.^2^, ^3^, ^4^ Given that there is a commercial assay available to assess the level of immune suppression when using cyclosporine in dogs, based on T‐cell cytokine expression, and given that many patients are receiving ≥1 immunosuppressive drugs in addition to cyclosporine, knowing the possible effects of these other medications on the expression of IL‐2 and IFN‐γ would be beneficial to interpreting the assay as well as enabling sound decision‐making regarding treatment recommendations.

When utilizing ΔCt values, mycophenolate mofetil, azathioprine and leflunomide did not have a detectable effect on expression of IL‐2 and IFN‐γ. Evaluation of the effects of oral administration of mycophenolate mofetil, azathioprine, and leflunomide on IL‐2 and IFN‐γ expression has not previously been performed in dogs. Our results suggest that suppression of T‐cell cytokine expression is not an important component of the mechanism of action of mycophenolate mofetil, azathioprine and leflunomide. Furthermore, our results suggest that the T‐cell cytokine pharmacodynamic assay would most likely be of minimal benefit for evaluating level of immunosuppression in dogs receiving any 1 of these 3 drugs and that, as has been reported in human medicine, concurrent administration of these medications would be unlikely to have a clinically relevant effect in interpretation of assay results in dogs receiving cyclosporine.

Prednisone and cyclosporine did detectably suppress the expression of IL‐2 and IFN‐γ, with cyclosporine markedly decreasing cytokine expression, and prednisone having a more moderate effect. When evaluating percent suppression by using the ΔΔCt method, the median percent of baseline activated samples for prednisone for IL‐2 and IFN‐γ, when comparing treatment values to baseline activated samples before treatment, was 23 and 16%, respectively (consistent with 77 and 84% suppression from baseline function, respectively). The median percent of baseline‐activated samples for cyclosporine for IL‐2 and IFN‐γ, when comparing treatment values to baseline‐activated samples before treatment, was 0.3% for both (consistent with 99.7% suppression from baseline function). The pharmacodynamic assay commercially available through the Mississippi State University Pharmacodynamic Laboratory currently utilizes activated T‐cell cytokine expression to titrate cyclosporine doses in dogs. In this current study, prednisone induced sufficient suppression of cytokine expression to be categorized in the “moderate suppression” category (50%‐80% immune suppression), a category that, for cyclosporine, would typically be insufficient to control severe systemic immune‐mediated disease. Cyclosporine, in contrast, induced profound suppression of cytokine expression, sufficient to be categorized in the “marked” suppression category (95%‐100% immune suppression), a level that is clinically associated with an increased risk of infection. Proposed mechanisms of action for glucocorticoids such as prednisone include inhibition of production and release of cytokines, impaired macrophage activity, impaired complement function, decreased antibody binding, inhibition of antibody production, decreased numbers of lymphocytes, and decreased migration of inflammatory cells into tissues.^1^ Given that 1 of the myriad of potential mechanisms of action of glucocorticoids is inhibition of cytokine production and release, it is not surprising that administration of prednisone had a detectable effect on IL‐2 and IFN‐γ expression. Our results suggest that when running the pharmacodynamic assay in clinical patients receiving both prednisone and cyclosporine, resultant suppression of cytokine expression could be because of the cyclosporine treatment, the prednisone treatment, or a combination of both.

Blood drug concentrations were measured in all dogs receiving cyclosporine, mycophenolate mofetil, or leflunomide. There were no readily available assays for measuring blood concentrations of prednisone or azathioprine at the time of this study. Samples were collected at the appropriate standard time points (peak for cyclosporine, and trough for leflunomide and mycophenolate mofetil) as recommended by the Auburn University Clinical Pharmacology Laboratory for clinical assays of drug concentrations, and processed and shipped according to the laboratory standard protocols. Although all dogs were administered similar drug dosages for these 3 medications, there was a wide variation in blood concentrations dog‐to‐dog for all 3 drugs. This observation emphasizes the fact that a single standard dose is not metabolized similarly in all patients, even in healthy dogs of a similar breed. For cyclosporine, the Auburn University Clinical Pharmacology Laboratory recommends a target peak blood concentration range from 1600 to 2400 ng/mL when treating immune‐mediated diseases. In this study, the highest peak blood concentration (3052 ng/mL) was almost double the lowest peak concentration (1672 ng/mL), despite both dogs having very similar inhibition of IL‐2 (1.4% of baseline versus 0.8% of baseline, respectively) and IFN‐γ (0.7% of baseline versus 0.5% of baseline, respectively). Mycophenolate mofetil similarly demonstrated marked variation in trough plasma mycophenolic acid concentrations between dogs administered similar dosages, with the highest trough concentration (5.8 μg/mL) being almost 20 times higher than the lowest trough concentration (0.3 μg/mL). For IL‐2, at both the highest and lowest observed mycophenolic acid concentrations, minimal effects on cytokine expression were observed, with the dog with the highest concentration demonstrating 123% of baseline IL‐2 values, and the dog with the lowest concentration demonstrating 91% of baseline values. Target plasma mycophenolic acid concentrations have not yet been established for dogs. The highest trough plasma concentration of teriflunomide observed, 123.2 μg/mL, was almost double that of the lowest (77.3 μg/mL), despite comparable oral doses of leflunomide. As with mycophenolic acid, for IL‐2, at both the highest and lowest observed teriflunomide concentrations, minimal effects on cytokine expression were observed, with the dog with the highest concentration demonstrating 66% of baseline IL‐2 values, and the dog with the lowest concentration demonstrating 138% of baseline values. Target plasma teriflunomide concentrations have not yet been established for dogs but, based on human data and clinical experience with canine samples submitted to the Auburn University Clinical Pharmacology Laboratory, a concentration greater than 16 μg/mL is considered to be a reasonable starting point for therapeutic response, and subjective clinical therapeutic responses have been observed in dogs with concentrations ranging from 5 to 45 μg/mL. Based on recommended target ranges, all of the dogs in this study exceeded target plasma teriflunomide concentrations, without a significant effect on cytokine expression. Our results therefore suggest that the mechanism of leflunomide immunosuppression, at least during the first week of drug administration, is independent of suppression of T‐cell cytokine expression.

We utilized GAPDH as a reference gene for all treatments because this is the gene that has been used in our cyclosporine assay. However, we understand that in gene expression studies, there is no single reference (housekeeping) gene that is suitable for all experimental conditions and each reference must be empirically validated for each treatment, even when monitoring expression of the same gene being evaluated, in the same cell type, of the same species. In this current study, while we did not perform a formal validation for each treatment, GAPDH expression was assessed across treatment groups and found to be comparable both before and after treatment, supporting GAPDH as an appropriate reference gene for this particular study. [Correction added on March 16, 2020, after first online publication: See Appendix S1 for correction details.]

This study used dogs of a single breed, and utilized healthy dogs rather than dogs with inflammatory or immune‐mediated diseases. Different results might have been attained in studies by using different breeds, or in diseased dogs. Although the effects of cyclosporine on IL‐2 and IFN‐γ expression are known to have reached maximal effect before 7 days of drug administration, the other drugs and their effects on cytokine expression over time have not been evaluated. It is possible that with a longer duration of administration, a detectable effect might have been seen with azathioprine, mycophenolate mofetil, and leflunomide, and a more pronounced effect might have been seen with prednisone. Although there was sufficient statistical power to detect differences in both IL‐2 and IFN‐γ expression before and after treatment with cyclosporine or prednisone, as well as detecting differences in expression between cyclosporine and prednisone treated dogs, it is possible that the sample size of 8 dogs did not provide sufficient power to detect an effect by the other drugs resulting in Type II errors.

In conclusion, cyclosporine and prednisone had a significant effect on expression of IL‐2 and IFN‐γ, with cyclosporine having a more pronounced effect than prednisone. Azathioprine, mycophenolate mofetil, and leflunomide did not have a significant effect on these 2 cytokines. At a cyclosporine dosage of 10 mg/kg PO every 12 hours, a significant and profound effect was seen in every dog. Prednisone had less pronounced and more variable effect on these cytokines. Blood drug concentrations were highly variable when dosing cyclosporine, mycophenolate mofetil, and leflunomide.

## CONFLICT OF INTEREST DECLARATION

Drs Archer, Mackin, and Thomason oversee the Pharmacodynamic Laboratory at the Mississippi State University College of Veterinary Medicine, which accepts samples from practitioners submitted for pharmacodynamic monitoring of cyclosporine treatment. Dr Boothe oversees the Clinical Pharmacology Laboratory at the Auburn University College of Veterinary Medicine, which accepts samples from practitioners submitted for therapeutic drug monitoring in dogs receiving cyclosporine or leflunomide.

## OFF‐LABEL ANTIMICROBIAL DECLARATION

The authors declare no off‐label use of antimicrobials.

## INSTITUTIONAL ANIMAL CARE AND USE COMMITTEE (IACUC) OR OTHER APPROVAL DECLARATIONS

Experimental and animal care protocols were approved by Mississippi State University IACUC. Mississippi State University is accredited by the Association for Assessment and Accreditation of Laboratory Animal Care.

## HUMAN ETHICS APPROVAL DECLARATION

Authors declare human ethics approval was not needed for this study.

## Supporting information


**Appendix S1.** Description of corrections.
