# Supplementary material for: Effects of oral administration of 5 immunosuppressive agents on activated T‐cell cytokine expression in healthy dogs
Source: J Vet Intern Med. 2020 Feb 13;34(3):1206–13. doi: 10.1111/jvim.15729 (PMC7255661; doi:10.1111/jvim.15729)
Supplement: Supplementary file 1 — Appendix S1. Description of corrections. [file JVIM-34-1206-s001.docx]

**Appendix S1.** Description of corrections.

*In Abstract, Background:*

“, and determining whether these different medications affect IL-2 expression would be useful when performing pharmacodynamic monitoring during cyclosporine therapy.” was added.

*In Abstract, Methods:*

Original:

**Methods**: Randomized, cross-over study. Dogs were administered each drug for 1 week, with a washout of at least 21 days. Activated T-cell expression of IL-2 and IFN-γ mRNA was measured by quantitative reverse transcription polymerase chain reaction. Drug concentrations in blood were measured for cyclosporine, mycophenolate, and leflunomide metabolites.

Revised:

**Methods**: Randomized, cross-over study comparing values before and after treatment, and comparing values after treatment among drugs. Dogs were administered each drug at standard oral doses for 1 week, with a washout of at least 21 days. Activated T-cell expression of IL-2 and IFN-γ mRNA was measured by quantitative reverse transcription polymerase chain reaction. Blood drug concentrations were measured for cyclosporine, mycophenolate, and leflunomide metabolites.

*In Section 2.4:*

Original:

A SuperScript III Platinum SYBR Green One-Step kit with Rox as a reference dye (Invitrogen, Grand Island, New York, Cat no. 11736-059) was used to quantify expression of the genes of interest (IL-2 and IFN-γ) and the expression of the housekeeping gene GAPDH.

Revised:

A SuperScript III Platinum SYBR Green One-Step kit with Rox as a reference dye (Invitrogen, Grand Island, New York, Cat no. 11736-059) was used to quantify expression of the genes of interest (IL-2 and IFN-γ) and the expression of the reference gene GAPDH.

*Figure 2:*

“% Suppression” was changed to “% of Untreated Activated Baseline” in both parts of the figure.

*In Section 4, paragraph 7:*

Original:

We utilized GAPDH as a housekeeping gene because this is the gene that has been used in our previous studies of T-cell cytokine expression, and is also the housekeeping gene that we use in our commercial assay. There is, however, not a single consistently recognized best housekeeping gene for use in dogs when evaluating whole blood. GAPDH has been used extensively in the literature for a housekeeping gene when evaluating canine whole blood, and in 1 publication was shown to be the 3rd most stable gene in canine whole blood by the Normfinder algorithm.^16^ Additionally, in this current study, GAPDH expression was compared across treatment groups and found to be comparable both before and after treatment, confirming GAPDH as an appropriate housekeeping gene for this study.

16. Chimura N, Shibata S, Kimura T, et al. Suitable reference genes for quantitative real-time RT-PCR in total RNA extracted from canine whole blood using the PAXgene™ system. *J VetMed Sci*. 2011;73(8):1101-1104.

Revised:

We utilized GAPDH as a reference gene for all treatments because this is the gene that has been used in our cyclosporine assay. However, we understand that in gene expression studies, there is no single reference (housekeeping) gene that is suitable for all experimental conditions and each reference must be empirically validated for each treatment, even when monitoring expression of the same gene being evaluated, in the same cell type, of the same species. In this current study, while we did not perform a formal validation for each treatment, GAPDH expression was assessed across treatment groups and found to be comparable both before and after treatment, supporting GAPDH as an appropriate reference gene for this particular study.
